# The first complete mitochondrial genome sequence of the leucosiid crab *Pyrhila pisum* (Arthropoda, Decapoda, Leucosiidae)

**DOI:** 10.1080/23802359.2017.1407717

**Published:** 2017-11-27

**Authors:** Yeong-Jun Park, Chang Eon Park, Byung Kwon Jung, Jerald Conrad Ibal, YeonGyun Jung, Sung-Jun Hong, Gun-Seok Park, Seok Hyun Lee, Hyun Sook Ko, Jae-Ho Shin

**Affiliations:** a School of Applied Biosciences, Kyungpook National University, Daegu, Republic of Korea;; b Department of Biomedical Engineering, The University of Texas at Austin, Austin, TX, USA;; c Department of Life Science, Silla University, Busan, Republic of Korea;; d Institute of Agricultural Science & Technology, Kyungpook National University, Daegu, Republic of Korea

**Keywords:** *Pyrhila pisum*, leucosiid crab, Leucosiidae

## Abstract

*Pyrhila pisum* is known as leucosiid crab. So far, there is no study about whole mitochondrial genome in Leucosiidae family. Here, we report first the complete sequence of the mitochondrial genome from *P. pisum*, which is composed of 15,516 base pair encoding 13 protein-coding genes, 22 transfer RNAs, two ribosomal RNAs, and A + T-rich region. The nucleotide composition of *P. pisum* was G + C: 25.5%, A + T: 74.5%, with a strong AT bias. In phylogenetic analysis using whole mitogenome, it was figured out that *P. pisum* was closely related to *Sesarma neglectum* but their family is different.

*Pyrhila pisum* is classified in Leucosiidae family according to morphological classification (Galil 2009). This crab mostly inhabits tidal flats, continental coastal zone, and subtropical coasts in Asia such as China, Japan, Taiwan, and Korea. The preferences of feeds are changed by time and sex but usually it prefers bivalves especially *Arcuatula senhousia* and *Venerupis philippinarum* (Kobayashi 2013). At present, this species is considered a threatened endangered species due to reduction of tidal flats (Kobayashi and Archdale 2017). In this study, we sequenced the mitochondrial genome of *P. pisum* and figured out phylogenetic relationship to other organisms through comparative genomics.

A sample of *P. pisum* was collected in Gangseo-gu, Busan, Korea (GIS: 35°04′47″N 128°52′36″E). The whole body specimen is registered under the voucher number NIBRGR0000099840 in National Institute of Biological Resources, Korea. The mitochondria were separated from 1 g of muscle tissue by differential centrifugation (Graham 2001). The mitochondrial DNA was extracted using QIAamp Tissue Kit (Qiagen, Valencia, CA). To amplify the mitogenome, specific PCR primers for Decapoda were used (Martin et al. 2016). Additionally, PCR primers based on conserved 16S rRNA gene and *cox*1 were designed and tried as well. PCR temperature for extension was gradually decreased than regular cycle in order to pcrevent PCR inhibition caused by A + T-rich region (Su et al. 1996). After purification of PCR amplicons, NGS library preparation and sequencing were performed using the Ion Torrent PGM platform following the manufacturer’s instructions (Life Technologies/Thermo Fisher Scientific, Waltham, MA). Whole mitochondrial genome assembly and annotation were proceeded using the CLC genomics workbench 8.5 (CLC Bio, Aarhus, Denmark). Protein-coding gene (PCGs), transfer RNA (tRNAs), and ribosomal RNA (rRNAs) were confirmed by NCBI Basic Local Alignment Search Tool (BLAST) and tRNA-scan 1.21 (Altschul et al. 1990; Lowe and Eddy 1997).

The assembled mitogenome was composed of circular DNA with 15,516 bp containing 13 protein-coding gene (PCGs), two ribosomal RNA (rRNAs), 22 transfer RNA (tRNAs) and A + T-rich region. In 13 PCGs, three kinds of start codon such as ATG, ATA and ATT were found. Most of PCGs contained TAA stop codon except *cox1*, *cox2*, *cox3*, and *cytb*. These four PCGs were terminated by incomplete stop codon such as T(aa) or TA(a) (Yamauchi et al. 2003; Hou et al. 2007; Zhang et al. 2016). The overall nucleotide composition was asymmetric (A: 37.28%, T: 37.20%, G: 10.22%, C: 15.30%) with a strong AT bias. The complete mitogenome of *P. pisum* was submitted to GenBank (accession no. NC030047).

The Phylogenetic tree of *P. pisum* and other crabs in Decapoda order was conducted using MEGA 6.0 by neighbour-joining method with 1000 replicate bootstrap (Tamura et al. 2013). The phylogenetic location of *P. pisum* was close to Grapsoidea super family (Figure 1). Since there are limited mitogenome information in family of Leucosiidae, more sequence reports are needed to classify of Decapoda order. This result emphasize that the complete mitogenome sequence of *P. pisum* can contribute to the phylogenetic knowledge of the genus *Pyrhila* as well as family Leucosiidae.

**Figure 1.**
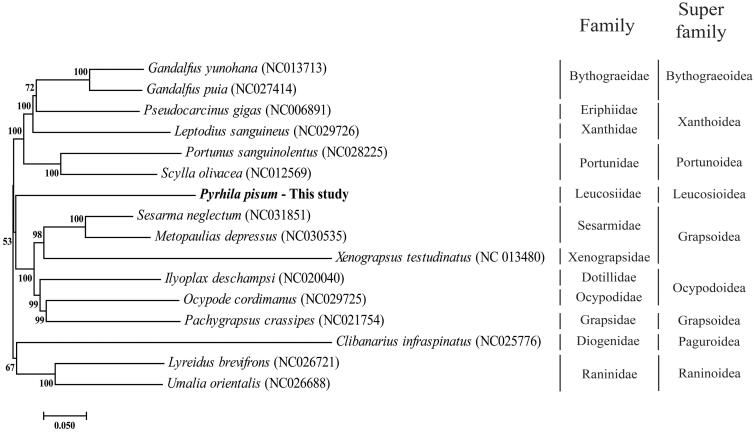
Phylogenetic tree of *P. pisum* with other crab in Decapoda order. The number in phylogenetic tree is bootstrap value. On the right side, vertical stick indicated specific family and super family of crab in Decapoda order. The GenBank accession numbers are indicated after the scientific name.
